# Do soil microbes and abrasion by soil particles influence persistence and loss of physical dormancy in seeds of tropical pioneers?

**DOI:** 10.3389/fpls.2014.00799

**Published:** 2015-01-13

**Authors:** Paul-Camilo Zalamea, Carolina Sarmiento, A. Elizabeth Arnold, Adam S. Davis, James W. Dalling

**Affiliations:** ^1^Smithsonian Tropical Research InstituteAncon, Panama; ^2^School of Plant Sciences and Department of Ecology and Evolutionary Biology, The University of ArizonaTucson, AZ, USA; ^3^Global Change and Photosynthesis Research Unit, United States Department of Agriculture – Agricultural Research ServiceUrbana, IL, USA; ^4^Department of Plant Biology, University of IllinoisUrbana, IL, USA

**Keywords:** Barro Colorado Island, germination cue, physical dormancy, pioneer plants, seed dormancy loss, seed persistence, soil seed bank

## Abstract

Germination from the soil seed bank (SSB) is an important determinant of species composition in tropical forest gaps, with seed persistence in the SSB allowing trees to recruit even decades after dispersal. The capacity to form a persistent SSB is often associated with physical dormancy, where seed coats are impermeable at the time of dispersal. Germination literature often speculates, without empirical evidence, that dormancy-break in physically dormant seeds is the result of microbial action and/or abrasion by soil particles. We tested the microbial/soil abrasion hypothesis in four widely distributed neotropical pioneer tree species (*Apeiba membranacea*, *Luehea seemannii*, *Ochroma pyramidale*, and *Cochlospermum vitifolium*). Seeds were buried in five common gardens in a lowland tropical forest in Panama, and recovered at 1, 3, 6, and 12 months after burial. Seed permeability, microbial infection, seed coat thickness, and germination were measured. Parallel experiments compared the germination fraction of fresh and aged seeds without soil contact, and in seeds as a function of seed permeability. Contrary to the microbial/soil abrasion hypothesis the proportion of permeable seeds, and of seeds infected by cultivable microbes, decreased as a function of burial duration. Furthermore, seeds stored in dark and dry conditions for 2 years showed a higher proportion of seed germination than fresh seeds in identical germination conditions. We determined that permeable seeds of *A. membranacea* and *O. pyramidale* had cracks in the chalazal area or lacked the chalazal plug, whereas all surfaces of impermeable seeds were intact. Our results are inconsistent with the microbial/soil abrasion hypothesis of dormancy loss and instead suggest the existence of multiple dormancy phenotypes, where a fraction of each seed cohort is dispersed in a permeable state and germinates immediately, while the impermeable seed fraction accounts for the persistent SSB. Thus, we conclude that fluctuations in the soil temperature in the absence of soil abrasion and microbial infection are sufficient to break physical dormancy on seeds of tropical pioneer trees.

## INTRODUCTION

Dispersal and site-specific seed survival play an important role in structuring plant communities by determining where seedlings can recruit (Hubbell et al., 1999; Harms et al., 2000). For pioneer species, germination from the soil seed bank (SSB) is an important determinant of species composition in gaps (Dalling et al., 1997, 2002), with seed persistence in the SSB allowing individuals to recruit in gaps that form up to several decades after seed dispersal (Dalling and Brown, 2009; Long et al., 2014). Seed persistence allows plants to germinate in favorable conditions (Ooi, 2012) while at the same time spreading the risk of reproductive failure through time (Long et al., 2014). Seeds can persist in soil as a result of dormancy, wherein seeds maintain physical or physiological barriers to germination, or as a result of quiescence, wherein seeds have no such barriers and germinate when conditions are favorable (Baskin and Baskin, 1989; Thompson, 2000; Dalling et al., 2011). Here, we use seeds of four physically dormant species of neotropical trees to understand factors that govern the breaking of physical dormancy in seeds in a seasonally moist tropical forest in Panama.

Physically dormant seeds are important components of the SSB in different ecosystems around the globe (Baskin and Baskin, 2000; Bradshaw et al., 2011; Moreira and Pausas, 2012; Benítez-Rodríguez et al., 2014; Ooi et al., 2014; Peguero and Espelta, 2014). These seeds possess a seed- or fruit-coat that is impermeable to water at the time of dispersal and prevents seed germination (Baskin and Baskin, 2000). Investment in physical defenses, such as thick seed coats in physically dormant seeds, may be the most effective means of prolonging seed persistence for seeds of pioneer trees in seasonal tropical forests (Dalling et al., 2011), and for weed species in temperate agricultural systems (Davis et al., 2008). In addition, physically dormant seeds, in which embryos are maintained in a dry state, are responsible for some of the most extreme examples of longevity recorded both in dry storage and in the soil (Thompson, 2000).

Strong evidence indicates that seeds with physical dormancy can break dormancy in response to environmental conditions in temperate (Thompson and Grime, 1983) and tropical regions (Pearson et al., 2002). Nonetheless, the germination ecology literature often speculates that physically dormant seeds lose dormancy as a consequence of microbial action and/or abrasion by soil particles (e.g., Went, 1955; Vazquez-Yanes and Orozco-Segovia, 1993; Lambers et al., 1998; Raven et al., 1999; Soriano et al., 2014; for a detailed list of references see Baskin and Baskin, 2000). For instance, five tropical species with impermeable seeds showed staggered germination (Soriano et al., 2014), leading the authors to suggest that the gradual loss of physical dormancy in these seeds could be a consequence of the action of soil microbes on the seed coat.

In turn, although physically dormant seeds cannot cycle between dormancy and non-dormancy, physically dormant seeds of some species of Fabaceae and Convolvulaceae can cycle between states of insensitivity and sensitivity to dormancy-breaking factors in the environment (Jayasuriya et al., 2009). Sensitivity cycling may be a common mechanism in temperate or tropical regions for short-lived seed banks that germinate seasonally, but seems improbable for pioneers that rely on temporally unpredictable recruitment sites (Baskin and Baskin, 2000).

If seeds that only possess physical dormancy are incapable of persisting in the soil once dormancy is broken, then it seems unlikely that microbial action or soil abrasion would determine the time course along which dormancy breaks. Although dispersal can be temporally decoupled from gap formation for tropical pioneer species, the timing of seed germination must be strongly coupled to gap occurrence for successful recruitment. In forests where canopy gaps recur unpredictably over decadal time scales, a dependency on microbial degradation would likely result in mostly fatal germination in the forest understory, with negative fitness consequences (Baskin and Baskin, 2000). In some species, however, seeds have a combinational dormancy type (i.e., physical in addition to physiological dormancy): the seed coat is water impermeable, and the embryo is physiologically dormant (Baskin and Baskin, 2007). If physical dormancy breakage is microbially induced in these cases, seeds could persist in a dormant state in the SSB after breaking physical dormancy. However, this raises the question of why seeds would express physical dormancy in the first place. Paulsen et al. (2013) suggested that physical dormancy is not only an adaptation to control germination, but might also protect seeds against predators by reducing olfactory cues.

When temperature and moisture conditions are favorable for seedling establishment in tropical forests, light quality and temperature fluctuations provide proximate cues for seed germination (Vazquez-Yanes and Orozco-Segovia, 1993; Pearson et al., 2002; Daws et al., 2006; Peguero and Espelta, 2014). However, for physically dormant seeds, microbial degradation of the seed coat and/or abrasion by soil particles has been widely proposed to be necessary to weaken seed protecting structures prior to responding to such cues. These suggestions arise because high temperatures characteristic of forest gaps are sometimes insufficient to induce germination (Vazquez-Yanes and Orozco-Segovia, 1993). Indeed under laboratory conditions, seeds with physical dormancy can require hot water treatments of 70–100°C to induce germination (Acuña and Garwood, 1987; Vazquez-Yanes and Orozco-Segovia, 1993, but see Daws et al., 2006), raising the possibility that the process of artificial dormancy-break differs from that which occurs under natural conditions where, at least in tropical forests gaps, the soil can reach a maximum of ∼50°C (Pearson et al., 2002). Thus an explicit experimental test is needed to distinguish proposals that either microbial action or physical abrasion by soil particles play a key role in seed coat rupture and subsequent germination of seeds with physical dormancy.

Here we measured germination responses, seed coat integrity and microbial colonization using fresh seeds of four widely distributed neotropical pioneer species (**Table 1**) in a seed burial experiment in the field, and in seeds that were stored in the laboratory. We tested the following hypotheses regarding loss of physical dormancy in tropical pioneer trees. (1) If seed coat abrasion and microbial degradation in the soil facilitates seed germination, then seed coats should become progressively more eroded with time in the soil, and penetration of seeds by bacteria and fungi should increase with time in the soil. (2) If seed germination in response to environmental cues is contingent on an initial loss of physical dormancy followed by seed quiescence (i.e., persistence of seeds in a non-dormant state), then the fraction of seeds that persist in the soil in a permeable state should increase with time in the soil. (3) If seeds germinate immediately from a previously physically dormant state, then seeds that are impermeable should be capable of germinating when provided with temperatures similar to those found in treefall gaps where they naturally recruit.

**Table 1:** Characteristics of focal species, including family, geographic distribution, number of maternal sources used in the burial experiment, fruiting period, and mass of fresh seeds at Barro Colorado Island, Panama.

Species	Family	Geographic distribution	Maternal sources	Fruiting period	Seed mass (mg)
*Apeiba membranacea* Aubl.	Malvaceae	Mexico–Brazil	6	February–March	18.9 ± 1.9
*Luehea seemannii* Triana and Planch.	Malvaceae	Mexico–Venezuela	5	February–April	1.9 ± 0.002
*Ochroma pyramidale* Urb.	Malvaceae	Mexico–Brazil	6	March–May	5.9 ± 0.7
*Cochlospermum vitifolium* Willd.	Bixaceae	Mexico–Brazil	8	February–April	25.6 ± 3.8


## MATERIALS AND METHODS

### STUDY SITE AND SPECIES

**FIGURE 1:**
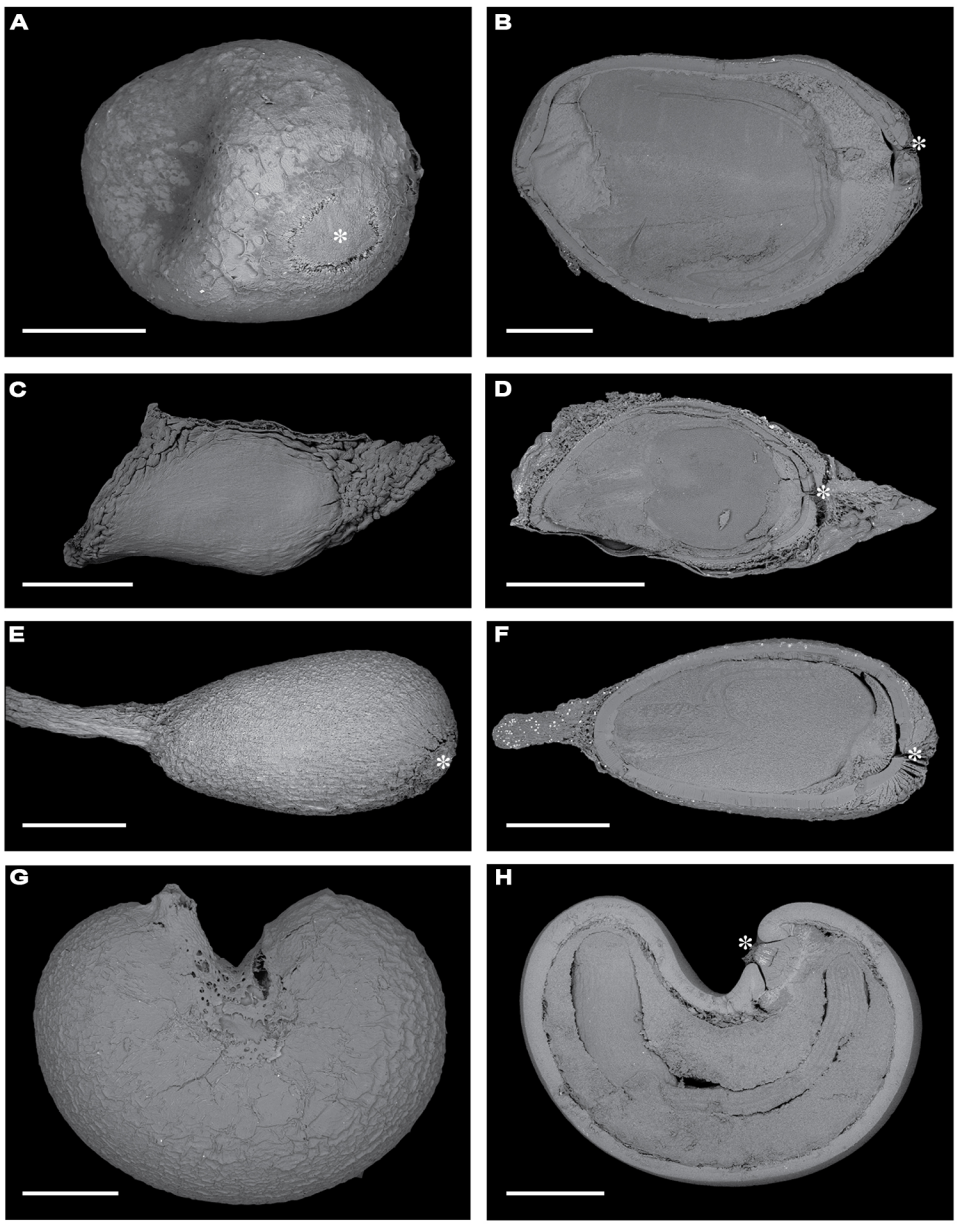
**Scanning electron microscopy images of the surfaces of fresh seeds (**A**, *Apeiba*; **C**, *Luehea*; **E**, *Ochroma*; G, *Cochlospermum*) and longitudinal sections of seeds (**B**, *Apeiba*; **D**, *Luehea*; **F**, *Ochroma*; **H**, *Cochlospermum*).** Scale bar = 1 mm. An asterisk highlights the chalazal area when visible.

The study was carried out in seasonally moist tropical forest at Barro Colorado Island, Panama (BCI: 9°10′N, 79°51′W). Rainfall on BCI averages 2,600 mm year^-1^, with a pronounced dry season from January to April (Windsor, 1990). The flora and vegetation of BCI have been described by Croat (1978) and by Foster and Brokaw (1982). Here, we used four species of pioneer trees whose seeds have physical dormancy (**Figure 1**). Three of them (*Luehea seemannii*, *Ochroma pyramidale*, and *Cochlospermum vitifolium*) are wind dispersed; the other (*Apeiba membranacea*) is dispersed by mammals (**Table 1**). All are common and widely distributed throughout the neotropics, and occur naturally at BCI (Croat, 1978; **Table 1**).

### COMMON GARDEN EXPERIMENT

From mid-February to mid-April 2012, ripe fruits were collected from the canopy of, or the ground beneath, at least five maternal trees of each of the four species at BCI or Gamboa (15 km southeast of BCI). Seeds were removed from fruits and cleaned manually to remove fruit pulp or cottony filaments. Clean seeds were air-dried at room temperature (∼22°C) in the dark for several days prior to use in burial experiments. To remove especially persistent pulp residues, seeds of *A. membranacea* were rinsed with 0.7% sodium hypochlorite for 2 min before burial.

From March to April 2012 we buried seeds in small mesh bags beneath the mature forest canopy in five 9 × 15 m common gardens on BCI. We used a randomized complete block design, with gardens spanning multiple soil types (Baillie et al., 2006; BCI soil map: http://strimaps.si.edu/webmaps/bcnm/). Gardens were located in the understory in sites that contained no adults of the study species within ≤20 m of garden edges. Prior to burial, seeds from all maternal sources of a single species were pooled, thoroughly mixed, and separated into burial units (BU) consisting of 45 seeds. Each BU was mixed with 10 g of sterile forest soil (i.e., previously autoclaved at 121°C for 2 h), enclosed in a nylon mesh bag (pore size = 0.2 mm), and covered with an aluminum mesh (pore size = 2 mm) to exclude seed predators. Four replicates consisting of four BUs per species were buried 40 cm apart at a depth of 2 cm below the soil surface, for a total of 16 BUs per species per garden.

### SEED PROCESSING AFTER RETRIEVAL FROM BU

Overall, 300 BUs (i.e., 94%) were successfully retrieved at 1, 3, 6, or 12 months after burial. After collection, seeds from each BU were rinsed with tap water and partitioned for tests of (i) germination (10 seeds per BU), (ii) microbial infection (10 seeds per BU), (iii) permeability (eight seeds per BU), and (iv) seed coat thickness (two seeds per BU). If we recovered fewer than 30 seeds per BU, seeds were partitioned as evenly as possible, prioritizing from most to least important: (i) germination, (ii) microbial culture, (iii) permeability, and (iv) seed coat thickness. To determine initial values for each trait, we used 600 fresh seeds per species from the original seed collection for germination (200 seeds), microbial culture (200 seeds), permeability (160 seeds), and seed coat thickness (40 seeds). Data on fresh seeds were collected from March to April 2012 and are presented in figures but not included in statistical analyses, which focus on how seeds lose dormancy after dispersal to the SSB.

For germination tests, seeds were placed in Petri dishes lined with paper towel, moistened with sterile distilled water, sealed with two layers of Parafilm®, and incubated for 6 weeks in a shadehouse in BCI under 30% full sun, high red: far-red irradiance (ca. 1.4), and ambient temperature (Pearson et al., 2002). The maximum temperature recorded on the germination bench was ∼38°C, similar to the temperature near the soil surface in large treefall gaps on BCI (Marthews et al., 2008), but lower than the maximum temperature recorded by Pearson et al. (2002) in large gaps (i.e., 52°C). Germination was defined as radicle protrusion and was recorded weekly. Permeable seeds or seeds that lost dormancy during the germination experiment swelled when imbibed, whereas dormant seeds did not. Thus hard seeds that did not germinate after 6 weeks were assessed for viability using the tetrazolium test (TZ; 2, 3, 5-triphenyl tetrazolium chloride; Peters, 2000). Ungerminated seeds scored as viable after TZ testing were considered dormant.

The proposed mechanism by which soil microbes break physical dormancy is through penetration of the seed coat (Vazquez-Yanes and Orozco-Segovia, 1993). We assessed whether microbes had penetrated seeds by evaluating microbial infection within seeds while eliminating microbes that were restricted to the seed surface. Seeds were surface-sterilized with successive immersion in 95% ethanol (10 s), 0.7% sodium hypochlorite (2 min) and 70% ethanol (2 min), allowed to surface-dry, and cut in half under sterile conditions (Gallery et al., 2007). For microbial isolation, one-half of each seed was placed in a unique 1.5 mL microcentrifuge tube containing 2% malt extract agar (MEA). After at least 4 months of incubation, microbial growth was macroscopically scored as zero (no growth) or one (growth); if microbial growth was detected, it was identified as fungal or bacterial based on culture morphology using a stereomicroscope.

To determine the extent of seed coat permeability, seeds were incubated in 0.1% (w/v) aqueous solution of Lucifer yellow CH potassium salt (hereafter LY; Biotium, Inc., CA, USA) for 48 h in the dark at room temperature (22°C). LY has a low molecular weight compared to other water-soluble fluorophores, making it especially useful for measuring seed permeability (Tieu and Egerton-Warburton, 2000). LY was removed by pipette after incubation, and seeds were rinsed twice in distilled water prior to being cut in half with a razor blade. Seed halves were examined using a Nikon Eclipse 600 microscope attached to a XX-V mercury lamp, with a Nikon B-2A fluorescent filter set (450–490 nm excitation/515 nm emission) and permeability was scored as zero (no LY on the endosperm) or one (LY on the endosperm).

For species in the Malvales with physically dormant seeds (including our four focal taxa), the palisade layer (hereafter called the seed coat) is the most important barrier to seed permeability. Dormancy-break in these species is associated with imbibition through the chalazal area of the seed (Baskin and Baskin, 1998). In physically dormant seeds of *A. tibourbou*, it has been shown that manual removal of the chalazal plug facilitated water uptake and seed germination (Daws et al., 2006). Thus, we used seed permeability as a measure of chalazal integrity, and measured seed coat thickness to determine whether seeds were subject microbial decay and/or physical abrasion. To measure seed coat thickness, seeds were cut in half under a dissecting scope and scanned using a Zeiss – Evo 40 vp scanning electron microscope (**Figure 1**). Mean seed coat thickness was determined from measurements at four random points for each seed image using ImageJ (http://rsbweb.nih.gov/ij/) and averaged for each seed.

### SEED AGING

To determine if changes in the fraction of germinable seeds over time was a response to conditions in the soil or simply a consequence of seed age, we set up an additional germination experiment. Seeds from the same seed lot used in the common garden experiment were stored in dry and dark conditions in an air-conditioned laboratory at 22°C for 2 years (i.e., until February 2014). Two hundred seeds per species were placed in Petri dishes lined with moist paper towel and sealed with Parafilm®, and incubated for 6 weeks in a shadehouse on BCI, using identical conditions as those used after burial. Germination was recorded every week for 6 weeks. *Cochlospermum* was not evaluated because seeds were not available from the original seed lot.

### TIME COURSE OF DORMANCY BREAKAGE

An additional germination experiment was used to determine the time course of dormancy-break under natural conditions for seeds shown previously to be impermeable. In April–May 2013, fresh seeds of *Apeiba* and *Ochroma* were collected from fruit and cleaned as described above prior to storage in dry and dark conditions at 22°C for up to 9 months. At the beginning of the experiment, seed permeability was assessed by individually weighing each seed, soaking each seed in 8 mL of distilled water for 24 h, and then measuring the conductivity of the soak water using an automatic seed conductivity analyzer (SAD-9000-S, Version 4.1.0, Soluciones Tecnológicas Globales, Argentina). Immediately after measuring conductivity, seeds were surface-dried with blotting paper and weighed again. Seeds were classified as permeable when electrical conductivity of the soak water was >10 μS and soaking yielded an increase of at least 30% in seed mass. Impermeable seeds showed no increase in mass or change in conductivity. Seeds were then sorted into three groups (25 seeds per species per group): (i) permeable seeds, (ii) impermeable seeds, and (iii) impermeable seeds treated with hot water (60°C for *Apeiba* and at 100°C for *Ochroma*) for 30 s to break dormancy (Acuña and Garwood, 1987).

Seeds were placed in Petri dishes lined with moist paper towel and sealed with Parafilm®. Petri dishes were placed on the upper bench of a greenhouse on the roof of the Smithsonian Tropical Research Institute’s Tupper Center in Panama City, Panama (100% full sun). The temperature of the paper towel inside each Petri dish was recorded using an infrared thermometer before harvesting seeds and after each harvest. We harvested a single seed per species, per treatment, two or three times a day over 5 days for a total of 36 *Apeiba* seeds (i.e., 12 permeable, 12 impermeable, and 12 impermeable – hot water treated) and 24 *Ochroma* seeds (i.e., eight permeable, eight impermeable, and eight impermeable – hot water treated). Individual seeds were evaluated immediately after collection for microscale responses to treatment (i.e., presence or absence of the chalazal plug, or cracks on the chalazal area) by examining the chalazal area with scanning electron microscopy (cold stage because seeds were processed while wet; Zeiss – Evo 40 vp scanning electron microscope; 125–175X).

### STATISTICAL ANALYSES

Using generalized linear mixed-effects models for each species, we analyzed changes in the proportions of (i) seeds that germinated and were dormant, (ii) seeds infected and non-infected by cultivable fungi, (iii) seeds infected and non-infected by cultivable bacteria, (iv) seeds that were permeable and impermeable, and (v) seed coat thickness, as a function of burial duration. Analyses were implemented in R version 2.15.3 (R Development Core Team, 2013) using the package *lme4* (version 1.0-5). For each combination of species and response variable, we coded each common-garden and its nested replicates as random effects and removal time as a fixed effect. If the response variable represented a proportion (e.g., permeable, impermeable seeds) over time, we used binomial error distributions to fit the generalized linear mixed-effect models. We tested whether there is an effect of removal time on the various responses by comparing models with and without removal time as explanatory variable using χ^2^ tests. We used restricted maximum likelihood estimation in the mixed-effect models, and two-tailed tests with a significance level set to 0.05. Although removal time is treated as a continuous variable in the statistical models it is presented as a categorical variable in figures to illustrate its effects on seed state and microbial infection using stacked bar charts.

## RESULTS

### SEED RECOVERY AT DIFFERENT TIME INTERVALS

**FIGURE 2:**
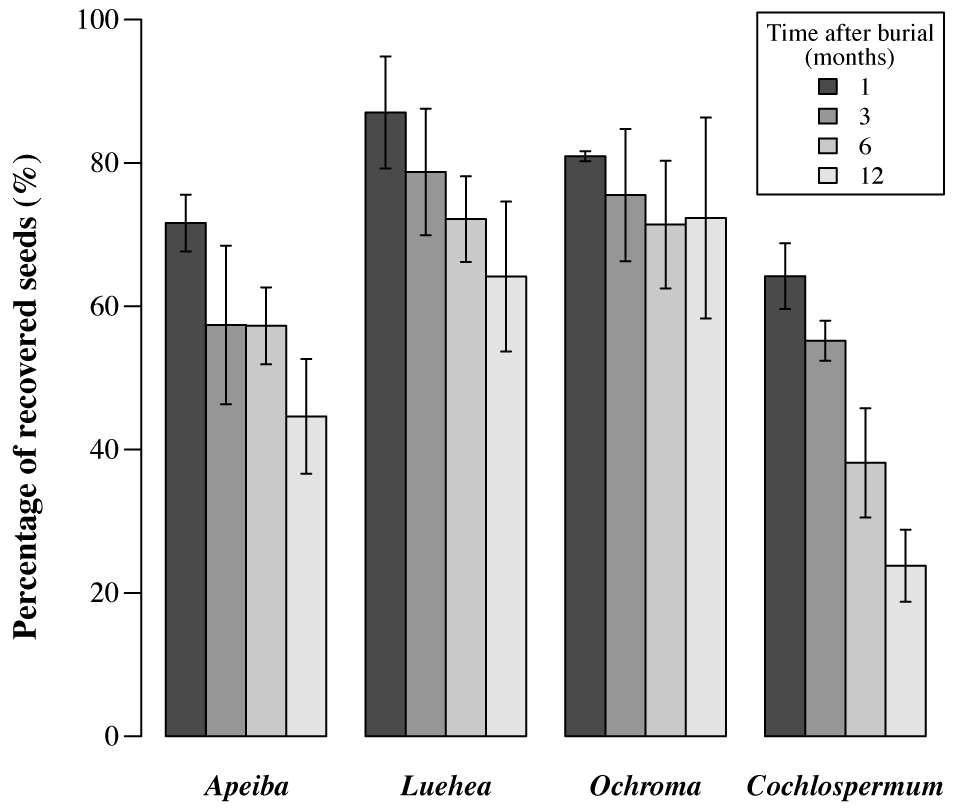
**Percentage of seeds retrieved at different time intervals.** Seed decay is equivalent to the total number of seeds buried (i.e., 100%), minus the number of seeds recovered successfully at each time. Error bars correspond to SD.

Here, we defined seed loss due to decay and/or fatal germination as the number of seeds initially buried, minus the number of seeds that was recovered intact at each retrieval time. After 12 months of burial, the seed recovery rate had declined to <30% in *Cochlospermum* and <50% in *Apeiba*, but remained >65% in *Ochroma* and *Luehea* (**Figure 2**). It is also important to highlight that for all the species included in this study, (i) the initial seed viability (i.e., fresh seeds) was high and ranged from 78% in *Apeiba* to 96% in *Cochlospermum*, (ii) after burial inviable seeds decayed rapidly and the majority of retrieved seeds were viable (viability ranged from 88% in *Apeiba* to 99% in *Ochroma*), and (iii) we found either germinated seedlings or fragments of seed coats inside the bags, at all retrieval times.

### SEED DORMANCY AND GERMINATION AS A FUNCTION OF BURIAL DURATION

**FIGURE 3:**
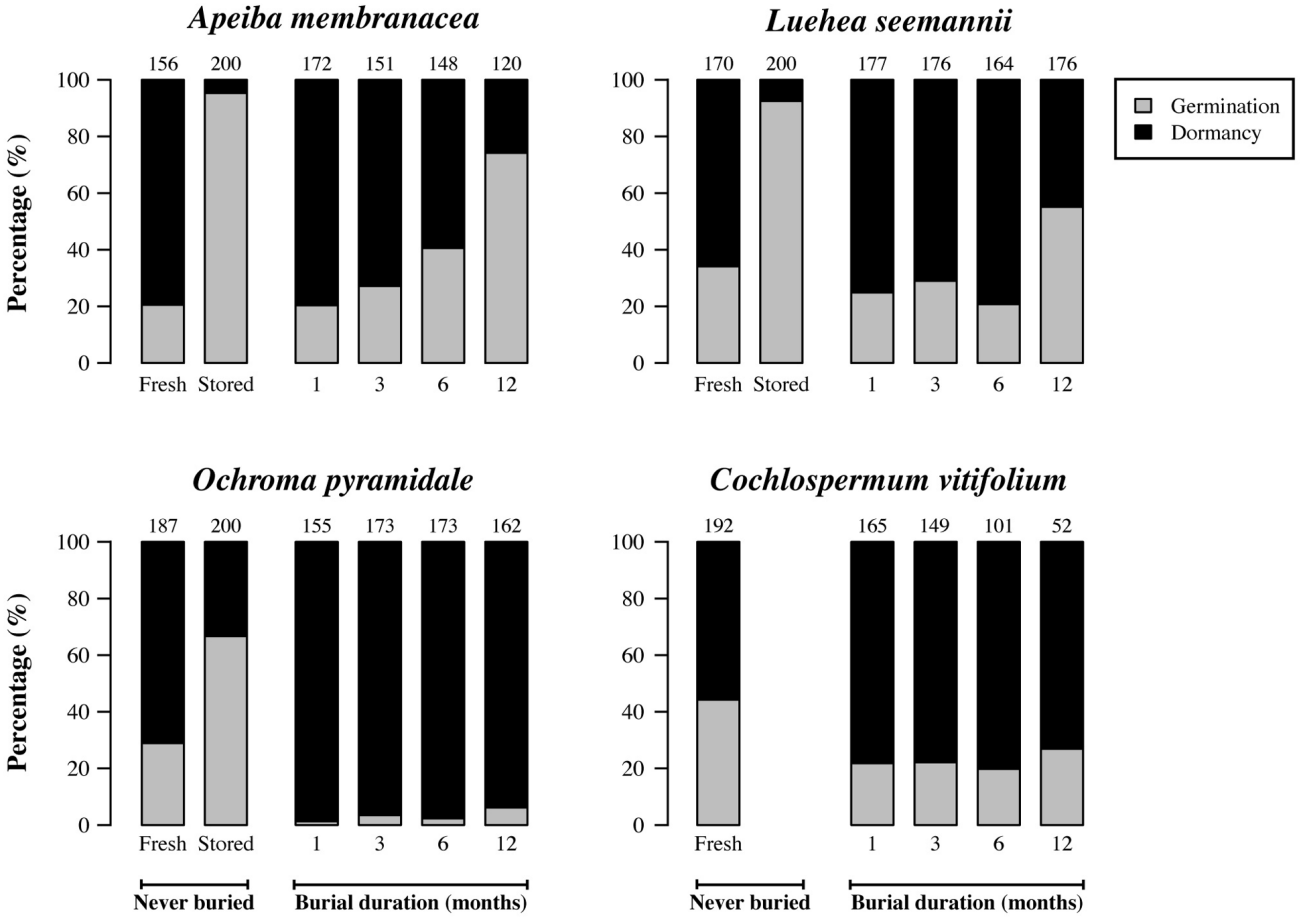
**Percentage of dormant seeds (black) and germinable seeds (gray) for fresh seeds; 2 years old, laboratory-stored seeds; and seeds successfully recovered at different time intervals after burial.** Numbers at the top of each bar represent the number of seeds examined for fresh (never buried), laboratory-stored (never buried), and buried seeds (i.e., the number of seeds retrieved from the common garden experiment and used for the germination experiment).

Seeds that had been stored in dry, dark, and cool conditions in the laboratory germinated more frequently than did fresh seeds (**Figure 3**). Germination in laboratory-stored seeds was 4.6, 2.3, and 2.7 times higher than in fresh seeds for *Apeiba*, *Luehea*, and *Ochroma*, respectively. For seeds retrieved from the soil, the fraction of seeds that germinated increased significantly over time for three species (**Figure 3**; **Table 2**). In *Cochlospermum*, the proportion of germinating seeds did not change over time (**Figure 3**; **Table 2**).

**Table 2:** Results of generalized linear mixed-effects models for each species.

	*Apeiba*		*Luehea*		*Ochroma*		*Cochlospermum*	
Factor	χ^2^	*p*	χ^2^	*p*	χ^2^	*p*	χ^2^	*p*
Germination: dormancy	95.04	<0.001	35.48	<0.001	4.73	0.029	0.23	0.632
Fungal: no fungal	3.25	0.071	8.71	<0.010	5	0.025	12.27	<0.001
Bacterial: no bacterial	9.48	<0.010	132.47	<0.001	141.54	<0.001	7.77	<0.010
Permeable: impermeable	29.6	<0.001	6.11	0.013	24.09	<0.001	3.57	0.059
Seed coat thickness	1.36	0.242	0.72	0.396	1.78	0.182	1.19	0.274

*Factors include the proportion of (i) seeds that germinated and were dormant, (ii) seeds infected and not infected by cultivable fungi, (iii) seeds infected and not infected by cultivable bacteria, (iv) seeds that were permeable and impermeable, and (v) seed coat thickness*.

*For all models *df* = 1. Please refer to the main text for more details on the models*.

### MICROBIAL INFECTION

**FIGURE 4:**
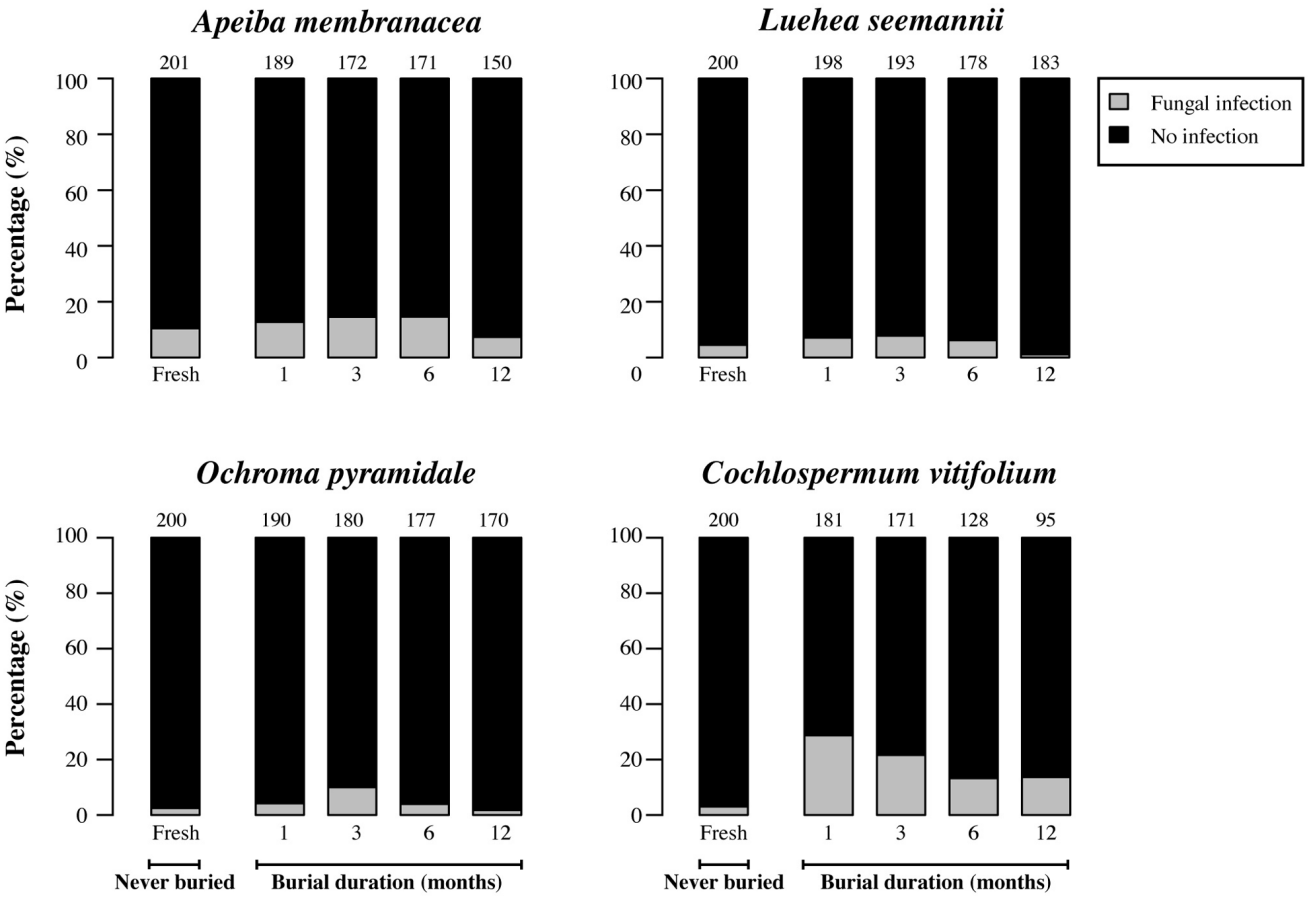
**Percentage of seeds infected by cultivable fungi (gray) and seeds without detectable infection (black) for fresh (unburied) seeds, and seeds successfully recovered at different time intervals following burial.** Numbers at the top of each bar represent the number of seeds examined for fresh seeds (never buried), and buried seeds (i.e., the number of seeds retrieved from the common garden experiment and used for assessing microbial growth).

**FIGURE 5:**
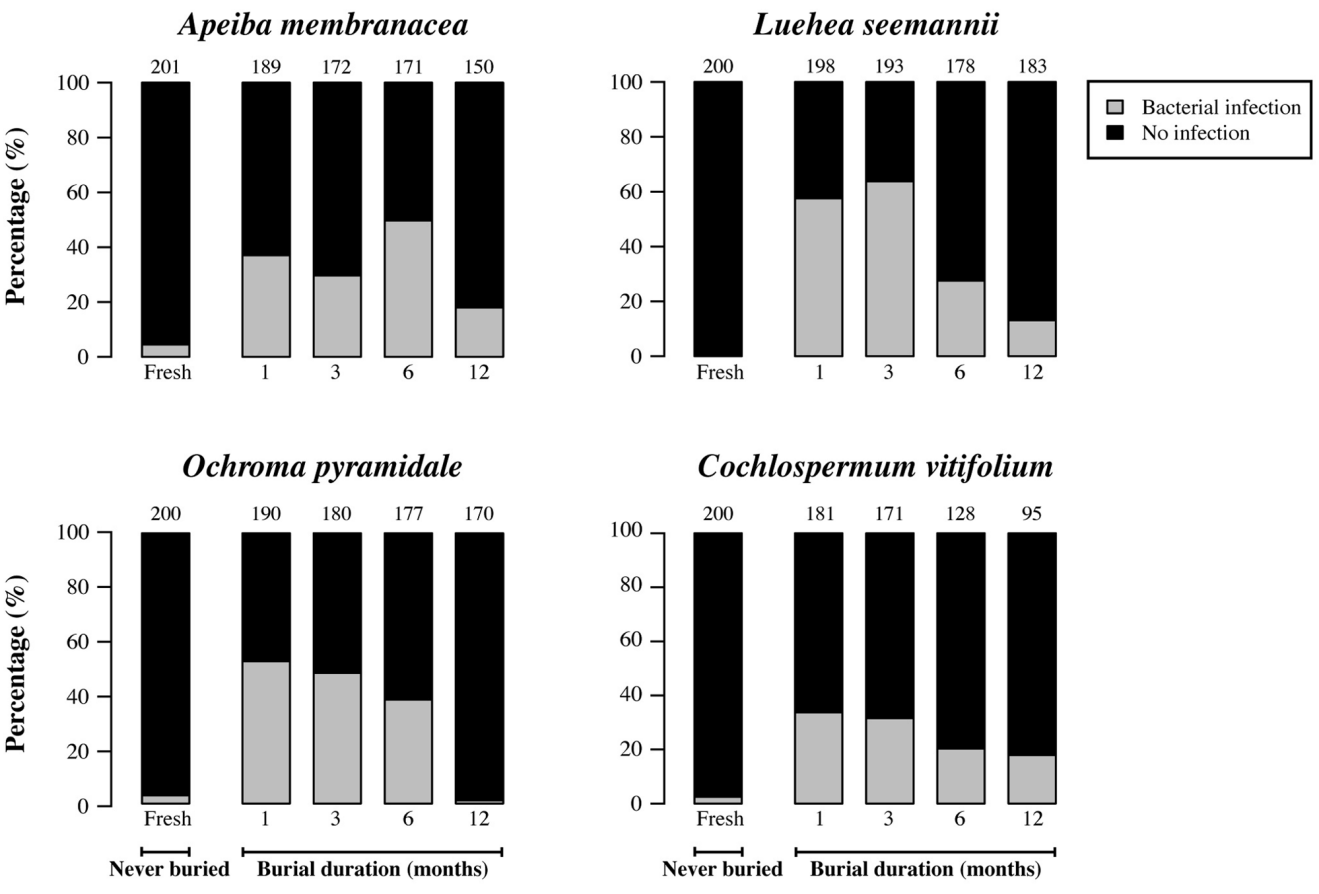
**Percentage of seeds infected by cultivable bacteria (gray) and seeds without detectable infection (black) for fresh (unburied) seeds, and seeds successfully recovered at different time intervals following burial.** Numbers at the top of each bar represent the number of seeds examined for fresh seeds (never buried), and buried seeds (i.e., the number of seeds retrieved from the common garden experiment and used for assessing microbial growth).

Fresh (unburied) seeds generally had infection rates that were 3.5–13 times lower than those of seeds that had been in soil for 1 month (**Figures 4** and **5**). However, the proportion of seeds infected by cultivable fungi decreased significantly with increasing burial duration for *Luehea*, *Ochroma*, and *Cochlospermum*, with a similar but non-significant trend observed for *Apeiba* (**Figure 4**; **Table 2**). Similarly, the proportion of buried seeds infected by cultivable bacteria decreased over time for all species (**Figure 5**; **Table 2**).

### SEED COAT INTEGRITY

**FIGURE 6:**
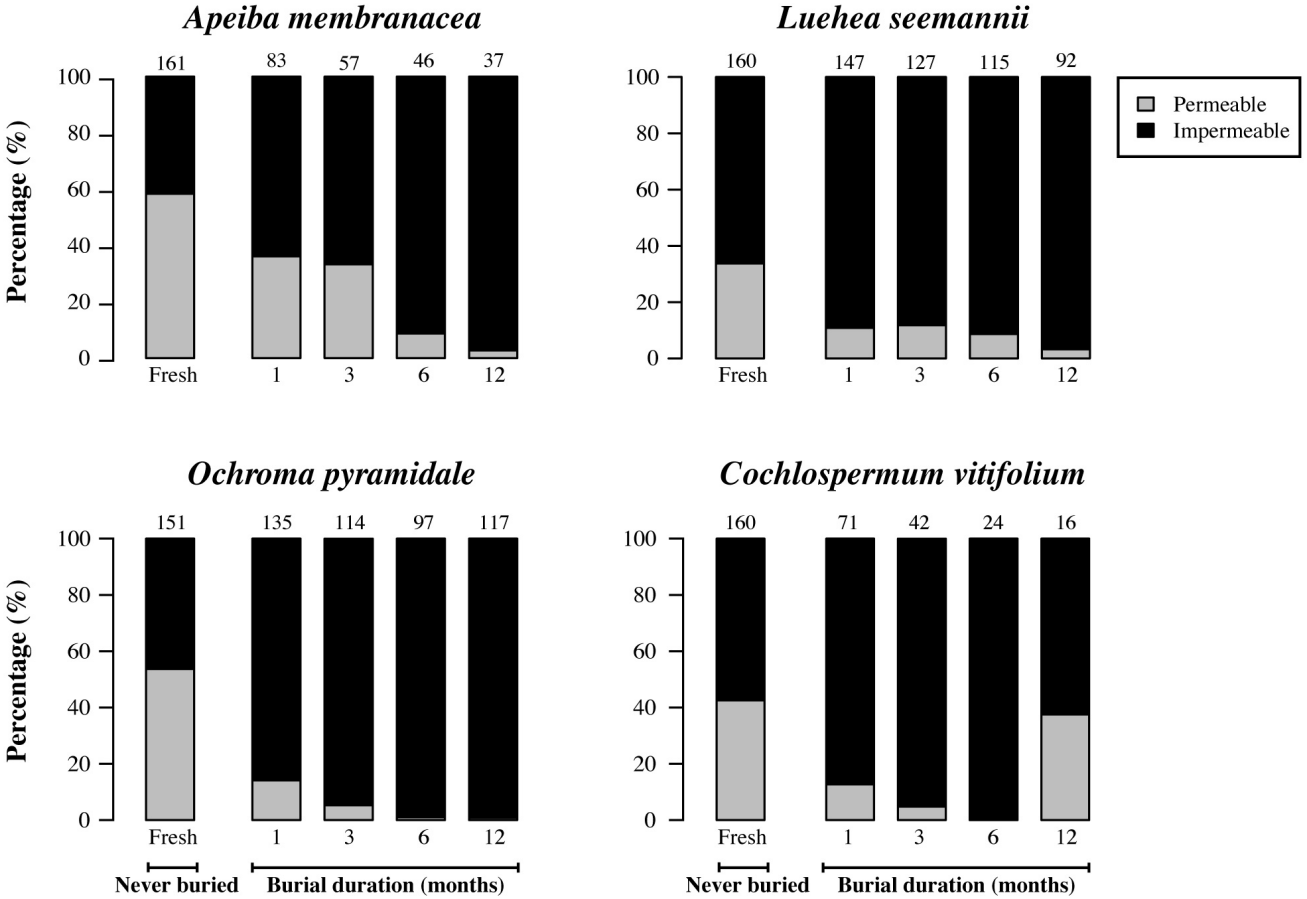
**Percentage of permeable seeds (gray) and impermeable seeds (black) for fresh (unburied) seeds, and seeds successfully recovered at different time intervals after burial.** Numbers at the top of each bar represent the number of seeds examined for fresh seeds (never buried), and buried seeds (i.e., the number of seeds retrieved from the common garden experiment and used to measure permeability).

After recovery from the soil, we found a significant decrease in the proportion of permeable seeds over time in *Apeiba*, *Luehea*, and *Ochroma*, but not in *Cochlospermum* (**Figure 6**; **Table 2**). No change in seed coat thickness was observed in any species over the burial period (**Table 2**). Average seed coat thickness is 122.7 ± 1.1 μm (average ± SE) for *Apeiba*, 47.7 ± 0.4 μm for *Luehea*, 125.6 ± 0.6 μm for *Ochroma*, and 178.4 ± 1.3 μm for *Cochlospermum*.

### TIME COURSE OF DORMANCY BREAKAGE

Seeds of two species (*Apeiba* and *Ochroma*) that were sorted into lots of permeable seeds, impermeable seeds, and impermeable seeds treated with hot water, and that were exposed to full sun, were assessed for evidence of chalazal plug lifting or cracks on the chalazal area using scanning electron microscopy.

**FIGURE 7:**
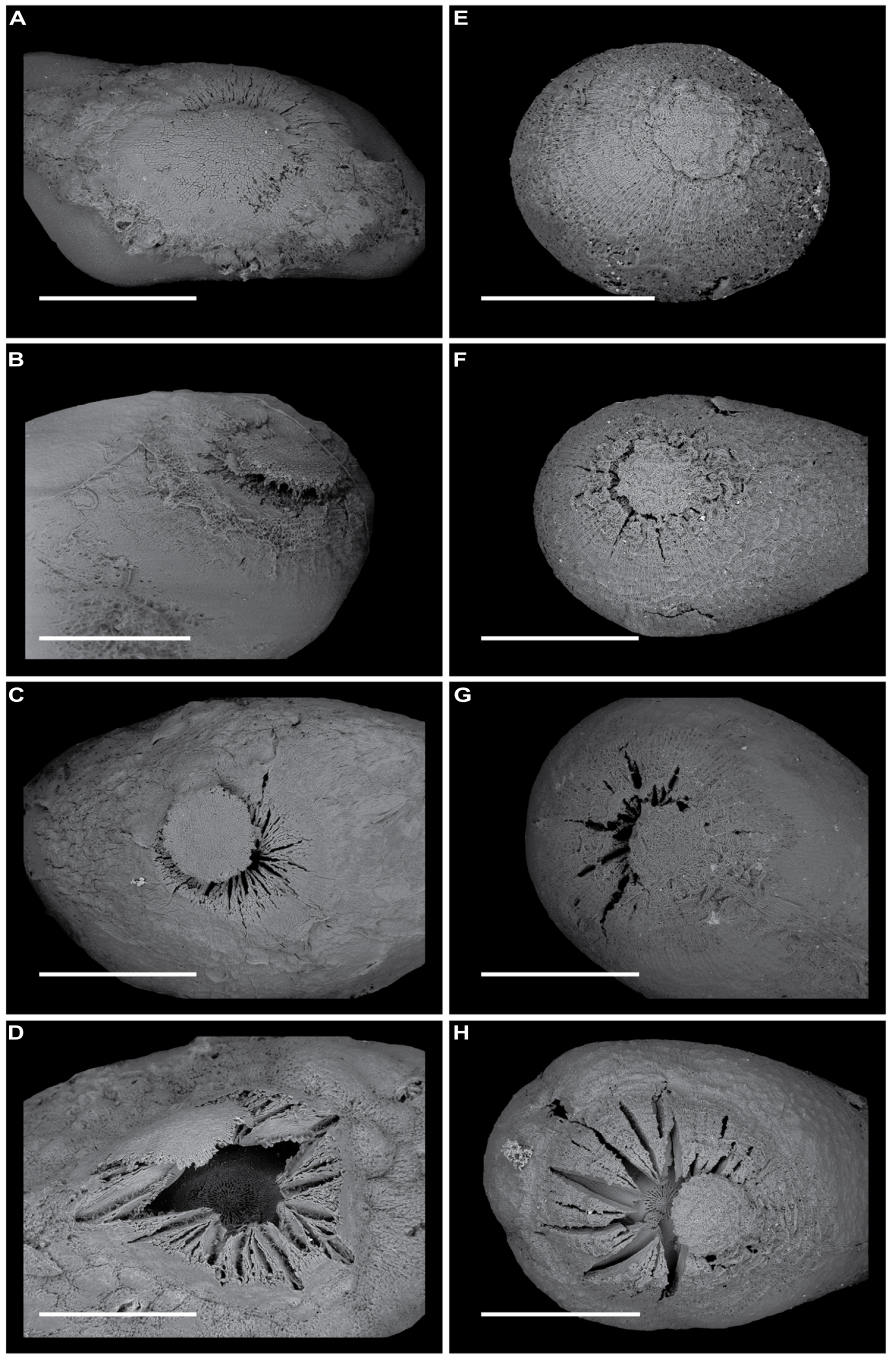
**Scanning electron microscopy images revealing the time course of chalazal plug breakage over 5 days for *Apeiba* seeds **(A–D)** and *Ochroma* seeds **(E–H)**.** At the onset of the experiment, seeds of *Apeiba* **(A)** and *Ochroma* **(E)** previously determined as impermeable had no evidence of cracks on the chalazal area, and the chalazal plug was attached to the seed surface. When seeds were exposed to full sun over 5 days, a gradual release of dormancy was observed as a progressive lifting of the chalazal plug in seeds of *Apeiba* **(B–D)** and *Ochroma* **(F–H)**. Scale bar = 1 mm.

At the onset of the experiment, all the permeable seeds of *Apeiba* and *Ochroma* either lacked a chalazal plug or showed cracks in the chalazal area (**Figures 7D,H**), whereas the surfaces of all impermeable seeds were intact. After treating impermeable seeds with hot water, 90% of *Apeiba* seeds and 50% of *Ochroma* seeds either lacked a chalazal plug, or had the chalazal plug lifted away from the seed coat (**Figures 7C,G**). Among impermeable seeds that were not treated with hot water, 33% of *Apeiba* seeds and 14% of *Ochroma* seeds showed a gradual release of dormancy over 5 days. This release of dormancy coincided with either (i) a lack of a chalazal plug or (ii) cracks in the chalazal area.

## DISCUSSION

Physical dormancy represents one important way in which plants from a wide variety of ecosystems can decouple the timing of seed dispersal from the availability of conditions conducive to successful germination and recruitment (Baskin and Baskin, 1998). Although the literature often alludes to the potential importance of microbial infection of seeds or abrasion of seed coats by soil particles in the loss of physical dormancy, other studies have found that physical dormancy in tropical pioneers can be broken by treatment with hot water (e.g., Vazquez-Yanes, 1974; Acuña and Garwood, 1987; Daws et al., 2006), mimicking one important cue consistent with presence in the forest gaps or edges needed for successful recruitment. The aim of our work was to test hypotheses regarding dormancy-break for physically dormant seeds.

Using seeds of four widely distributed neotropical pioneer species in a seed burial experiment, and in a germination experiment coupled with imaging of seeds by scanning electron microscopy, we found no evidence for an important role of infection by fungi or bacteria, or soil abrasion of seed coats, in influencing germination or dormancy-break. The proportion of permeable seeds generally decreased as a function of burial duration in soil, rather than increasing. Seeds that were confirmed to be impermeable were capable of germinating when provided with temperature fluctuations similar to those found in treefall gaps, reflecting release or dislocation of the chalazal plug (Daws et al., 2006).

### TEMPORAL CHANGES IN SEED GERMINABILITY AND PERMEABILITY

With the exception of *Cochlospermum*, we found that the proportion of buried seeds that were germinable increased through time, consistent with earlier observations in the literature that prompted speculation that soil abrasion or microbial action weakens seed coats. However, we also observed that the proportion of seeds that were permeable decreased, either when comparing seeds that had been buried for increasing periods of time, or when comparing fresh and buried seeds. The existence of a large fraction (30–60%) of seeds that are dispersed in a permeable state suggests the existence of multiple phenotypes among apparently identical fresh seeds. The decrease in the fraction of permeable seeds after burial is likely explained either by the decay of permeable seeds or by fatal germination, where seedlings died inside the mesh bag. In either case, seed loss reduced the number of remaining seeds, and enriched the proportion of impermeable seeds.

Results from the comparison of fresh and lab-stored seeds were also inconsistent with the hypothesis of microbial release from dormancy. Seeds stored in the lab for 2 years remained mostly impermeable, indicating that dry storage alone did not break dormancy; however, lab-stored seeds showed two- to fourfold higher germination than fresh seeds. These results indicate that it becomes easier for impermeable seeds to break dormancy as they age. This result is consistent with studies of physically dormant seeds in temperate and subtropical systems. For instance, Galíndez et al. (2010) found that after 4 years of dry storage, a high proportion of *Collaea argentina* and *Abutilon pauciflorum* seeds were more sensitive to dormancy-breaking treatments. Seed aging is widely described as the physiological process in which seeds lose vigor and may eventually die (Long et al., 2014); however, Mohamed-Yasseen et al. (1994) suggested that aging does not in itself necessarily cause death, but decreases resistance to stress. In the case of Malvaceae, the water gap is described as the chalazal oculus, an area occluded by a plug-like structure formed by water-impermeable sclerenchyma cells (Gama-Arachchige et al., 2013). Our findings suggest that the cells surrounding the chalazal plug are aging, making seeds more sensitive to temperature fluctuations that open the water gap and break dormancy.

At the time of dispersal, a large fraction of seeds in a given maternal seed cohort are already permeable, revealing the existence of multiple seed phenotypes (**Figure 6**). We found that high initial rates of seed permeability resulted in high initial germination in fresh seeds. Seed polymorphisms in species with physically dormant seeds have been previously reported on the basis of color, weight, or size (Kelly et al., 1992; Wei et al., 2007; Baskin et al., 2008). In addition, it has been recently shown that apparently identical fresh seeds can differ in permeability (Paulsen et al., 2013). Paulsen et al. (2013) showed that impermeable and permeable seeds of *Robinia pseudoacacia* and *Vicia sativa* were readily found by rodents when shed from the plant onto the soil surface and that the probability of predator escape during re-caching was higher for impermeable than for permeable seeds. They argued that the production of permeable seeds represents a payment for dispersal services provided by scatter-hoarding rodents, suggesting that physical dormancy has evolved to hide seeds from mammalian predators. In lowland and montane forest in Panama, however, Fornara and Dalling (2005) found that most of post-dispersal seed removal in six pioneer species (including two species used in this study) is attributed to litter ants, with no evidence of vertebrate seed removal. As an alternative hypothesis for the existence of multiple seed phenotypes in pioneer species, we propose that initial permeability is advantageous in allowing immediate germination of seeds dispersed directly into gaps, while impermeable seeds dispersed to the understory persist in the SSB (Brokaw, 1987; Pearson et al., 2003).

### SEED MICROBIAL INTERACTIONS AND ABRASION BY SOIL PARTICLES

If microbial infection causes a gradual loss of physical dormancy, we would expect an increase in microbial infection of seeds as a function of burial duration. However, our results show the opposite: an increase in the fraction of seeds capable of breaking physical dormancy was associated with a smaller fraction of seeds yielding cultivable microbes. In addition, the fraction of fresh seeds yielding microbes was consistently lower than that of buried seeds, indicating that microbial colonization occurred in the soil rather than during seed development. Collectively, these observations suggest that soil microbes are not the cause of physical dormancy loss. However, it is important to highlight that seeds of tropical pioneer species are frequently infected by fungi (Dalling et al., 1998; Gallery et al., 2007, 2010; Olvera-Carrillo et al., 2009), and microbial interactions in the soil are important in shaping recruitment patterns in tropical forests (e.g., Augspurger, 1984; Dalling et al., 1998; Gallery et al., 2010; Bagchi et al., 2014). In addition, it is also possible that fungal infection may trigger or enhance germination of physiologically dormant seeds (e.g., *Opuntia* spp. or dust seeds of myco-heterotrophic orchids that do not need forest gaps for recruitment; Bidartondo and Read, 2008; Olvera-Carrillo et al., 2009; Delgado-Sánchez et al., 2010, 2013).

We predicted that an extended length of time in soil would erode the seed coat, therefore making the seeds permeable. In turn, if soil abrasion triggers physical dormancy-break, we would expect an increase in permeability coupled with a decrease in seed coat thickness as a function of burial duration. Our results indicate instead that the proportion of permeable seeds decreases over time while seed coat thickness does not vary. Since all the permeable seeds imaged in this study either lacked a chalazal plug or showed cracks in the chalazal area, the increase in the fraction of impermeable seeds with time in soil suggests that all the recovered seeds that were impermeable had the chalazal area intact. Given that loss of dormancy in physically dormant seeds is irreversible (Jayasuriya et al., 2009), dormancy-break and subsequent germination should be linked tightly with suitable conditions for plant establishment (i.e., gap formation). Thus, species that need forest gaps to recruit are not expected to show sensitivity cycling in physical dormancy (*sensu* Jayasuriya et al., 2009) because gap formation is an unpredictable event in the forest, lacking strong seasonality (Brokaw, 1985).

### VISUALIZING DORMANCY-BREAK UNDER NATURAL TEMPERATURE CONDITIONS

Elevated soil temperature in gaps, associated with high direct solar radiation inputs, act as a mechanism triggering germination in tropical pioneer species with large seeds (i.e., seed mass > 2 mg), including the species in this study (Vázquez-Yanes and Orozco-Segovia, 1982; Pearson et al., 2002). Daws et al. (2006) found that hot water treatment of *A. tibourbou* seeds breaks physical dormancy by removing the chalazal plug, thus facilitating water uptake. Furthermore, Vazquez-Yanes and Orozco-Segovia (1993) suggested that many plant species, including *O. pyramidale*, have seeds with a suberized layer and impermeable seed coats that can be broken by heat, allowing water to penetrate the seed and thus breaking physical dormancy. They also suggested that some seeds need temperatures no higher than 45°C to become permeable to water. However, no direct observations exist of the process of dormancy-break under natural conditions for seeds shown previously to be impermeable.

Seeds of all four species included in this study have a chalazal plug (see **Figures 1** and **7**). When seeds were exposed to full sun in a greenhouse, we saw a gradual release of dormancy in seeds of *Apeiba* and *Ochroma* that were previously determined to be impermeable. As has been shown using a hot water treatment (Daws et al., 2006), these natural fluctuations in temperature resulted in a progressive lifting of the chalazal plug (**Figure 7**). The maximum temperature recorded in the germination experiment was 44.6°C, consistent with the suggestion that dormancy can be broken with temperatures ∼45°C (see Daws et al., 2006). Taken together, our results – along with those of Pearson et al. (2002) and Daws et al. (2006) – suggest that synchronous germination in forest gaps might best be explained by high temperature fluctuations between day and night, which promote the lifting of the chalazal plug in previously impermeable seeds.

### DORMANCY-BREAK AS AN AXIS OF NICHE DIFFERENTIATION IN TROPICAL PIONEERS

Although our results are consistent with previous reports showing that the species included in this study have seeds that persist for >1 year in the SSB (Dalling et al., 1997; Pearson et al., 2002; Soriano et al., 2014), we found that the proportion of decaying seeds, or the proportion of seeds remaining dormant after being subjected to germination cues, varied among species. The rate at which the proportion of dormant seeds decreases over time is likely linked to availability of recruitment sites for each species and may reflect differentiation in gap size requirements among pioneer species (Brokaw, 1987; Pearson et al., 2003). We also found that *Ochroma* displayed a very low rate of dormancy loss over time (i.e., a consistently small proportion of seeds germinating after exposure to favorable germination conditions) compared to *Apeiba* and *Luehea*. These results suggest that there may be a trade-off between the ability to break dormancy and the ability to persist in the SSB. *Ochroma* seeds are known to persist for long periods in the SSB and only recruit in very infrequent, large canopy gaps or at forest edges (Vazquez-Yanes and Orozco-Segovia, 1993).

Seed persistence and dormancy traits also may reflect variation in habitat conditions across the wider geographic range of species. For instance, Soriano et al. (2014) found that the germinable fraction of seeds in *Cochlospermum* increases after burial for some months in the soil of the Chamela dry forest in Mexico (although the amount of time the seeds spent in the soil is not specified by the authors, it is presumably more than a year), but in this study we did not find differences in the fraction of germinable seeds in *Cochlospermum* over 12 months of burial. *Cochlospermum* is often found in disturbed and early successional tropical dry forests from Mexico to northern South America (Bawa and Frankie, 1983). It is rare in the seasonally moist forest in Barro Colorado National Monument, occurring only in forest edges (Croat, 1978). *Cochlospermum* does not occur in wet forest and when present in seasonally moist forest, it is often associated with areas impacted by human disturbance, rather than natural forest gaps (Dalling and Zalamea, personal observation). Most dry forest species are deciduous for a few months during the dry season; it is possible that in these forests, *Cochlospermum* seeds do not need gaps for recruitment but instead recruit annually beneath leafless forest canopies at the onset of the wet season. It is also possible that seeds of *Cochlospermum* are adapted to colonize dry areas in which soils are often burned by fires and characterized by low soil moisture, high light availability, and high temperature, as suggested for *Ochroma* seeds (see Vazquez-Yanes, 1974).

## CONCLUSION

**FIGURE 8:**
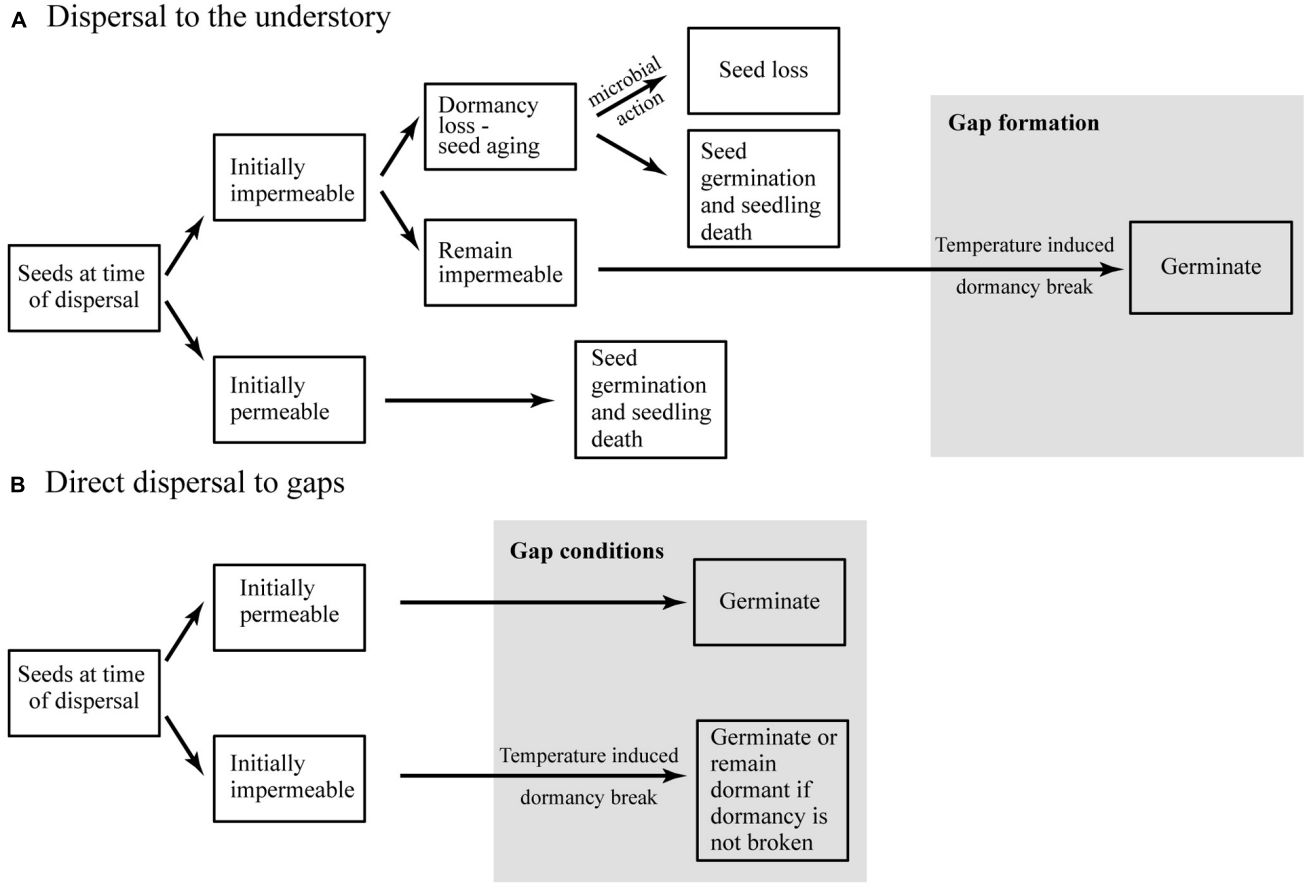
**Conceptual model summarizing seed persistence and germination in tropical forest species with physically dormant seeds dispersed to (**A**) the understory or (**B**) gaps**.

Germination requirements are likely to be under strong selective pressure because plant recruitment depends on successful emergence under conditions favorable for subsequent survival and growth. Based on our results, we conclude that fluctuations in the soil temperature in the absence of soil abrasion and microbial infection are sufficient to break physical dormancy (**Figure 8**). In this conceptual model we also propose that, at the time of dispersal, a large fraction of seeds of all species are permeable, resulting in a high initial germination rate. This suggests the existence of a seed dormancy polymorphism, whereby permeable seeds dispersed to gaps can germinate and recruit immediately, while impermeable seeds dispersed to the understory can persist in the SSB (**Figure 8**).

## Conflict of Interest Statement

The authors declare that the research was conducted in the absence of any commercial or financial relationships that could be construed as a potential conflict of interest.
